# Tissue clearing may alter emission and absorption properties of common fluorophores

**DOI:** 10.1038/s41598-022-09303-9

**Published:** 2022-04-01

**Authors:** Farsam Eliat, Rebecca Sohn, Henrik Renner, Theresa Kagermeier, Stefan Volkery, Heike Brinkmann, Nils Kirschnick, Friedemann Kiefer, Martha Grabos, Katharina Becker, Ivan Bedzhov, Hans R. Schöler, Jan M. Bruder

**Affiliations:** 1 Max Planck Institute for Molecular Biomedicine, Röntgenstr. 20, 48149 Münster, Germany; 2 University of Münster, Schlossplatz 2, 48143 Münster, Germany; 3 Embryonic Self-Organization research group, Max Planck Institute for Molecular Biomedicine, Röntgenstr. 20, 48149 Münster, Germany; 4 University of Münster, European Institute for Molecular Imaging, Waldeyerstraße 15, 48149 Münster, Germany

**Keywords:** High-throughput screening, Imaging, Microscopy

## Abstract

In recent years, 3D cell culture has been gaining a more widespread following across many fields of biology. Tissue clearing enables optical analysis of intact 3D samples and investigation of molecular and structural mechanisms by homogenizing the refractive indices of tissues to make them nearly transparent. Here, we describe and quantify that common clearing solutions including benzyl alcohol/benzyl benzoate (BABB), PEG-associated solvent system (PEGASOS), immunolabeling-enabled imaging of solvent-cleared organs (iDISCO), clear, unobstructed brain/body imaging cocktails and computational analysis (CUBIC), and ScaleS4 alter the emission spectra of Alexa Fluor fluorophores and fluorescent dyes. Clearing modifies not only the emitted light intensity but also alters the absorption and emission peaks, at times to several tens of nanometers. The resulting shifts depend on the interplay of solvent, fluorophore, and the presence of cells. For biological applications, this increases the risk for unexpected channel crosstalk, as filter sets are usually not optimized for altered fluorophore emission spectra in clearing solutions. This becomes especially problematic in high throughput/high content campaigns, which often rely on *multiband* excitation to increase acquisition speed. Consequently, researchers relying on clearing in quantitative multiband excitation experiments should crosscheck their fluorescent signal after clearing in order to inform the proper selection of filter sets and fluorophores for analysis.

## Introduction

### Volume imaging of 3D tissues is challenging

Understanding the spatial complexities of the three-dimensional arrangement of cells in tissues has always been a demanding task. Traditionally, tissues have been embedded and cut in serial sections, granting two-dimensional tools and techniques access to three- dimensional samples^1^. Common techniques like volume imaging, including confocal and light sheet microscopy, now routinely enable accurate molecular and structural analysis of intact 3D tissues^2^, even live^3^. However, the optical analysis of large cellular aggregates still poses challenges. Illuminated from a well-defined single direction, cells, organelles, membranes, and molecules reflect light in all directions causing light-scattering. Moreover, cells and cell compartments interact with light, reducing its velocity through the tissue to varying degrees, a phenomenon measured as the refractive index (RI). Simply put, a high density of molecules causes a long delay in the propagation of light, thus leading to a high RI. However, the RI depends not only on the tissue’s density, but also on its capability to absorb light. Some tissue compartments absorb more incoming light than others with high absorption contributing to a high RI. Light changes direction at interfaces with different RI and thus, the heterogeneity in the RIs between different cellular compartments and tissue regions further contributes to light-scattering. Consequently, all wavelengths of visible light are scattered causing a milky, even opaque appearance. Therefore, few biological tissues are translucent and visibility of cellular details is often limited to outer tissue layers of larger aggregates^4,5^.

### Tissue clearing enables optical analysis of intact 3D tissues with single cell resolution

Over the years, a variety of clearing approaches have helped reduce tissue opacity. Despite different chemical approaches, they all aim to reduce the amount of light scattering. They remove highly reflective and absorbent components and unify the refractive indices of different regions, by removing, replacing, or modifying different tissue compartments^6^. In this way, tissues ranging from 100 µm up to several centimetres become virtually transparent^7^. Various groups have optimized clearing for use with skin and soft tissue^8^, neuronal tissue^9–11^, liver tissue^12^, musculoskeletal tissue, bones^13^, for large scale 3D tissue aggregates (organoids)^14,15^, and even whole organisms^16,17^. The different clearing techniques can be grouped into families based on their chemistry: organic solvents, high refractive index aqueous solutions, hyperhydrating solutions, and tissue transformation reagents^18^, for a comprehensive review, see^19^.

### Many new arising clearing methods are based on the Benzyl Alcohol/Benzyl Benzoate (BABB)-approach

First introduced in 1914^20^, Benzyl Alcohol/Benzyl Benzoate (BABB) is the basis for many subsequent common clearing techniques and inspired FluoClearBABB^11^, 3DISCO^21^, iDISCO^22^ and uDISCO^23^.

The organic solvents extract highly scattering lipids, replace water by high RI agents, and thus homogenize the RI of samples to a high value above 1.5^24^. BABB-based clearing is a robust method to improve optical transparency of different tissue types, but the dehydration process also causes substantial tissue shrinkage^5,6^.

To avoid tissue shrinkage, other clearing techniques replaced the organic solvent BABB with hyperhydrating solutions like urea (Scale)^25^. In contrast to BABB-based clearing, this method partially denatures and hydrates even hydrophobic regions of the tissue. Thus, the refractive index of the sample is homogenized to a low value below 1.5. The addition of amphipathic glycerol (ScaleA2) or dehydrating sorbitol (ScaleS) further reduces or even prevents hydration-induced tissue expansion^26^.

### Clearing can modify the emission spectra of DAPI and Alexa Fluor secondary antibodies and thus may exacerbate fluorescent channel cross talk

While working with optically cleared samples, we noticed that some clearing techniques including both organic-solvent-based BABB, iDISCO, PEGASOS, and aqueous CUBIC and Scale protocols change the emission spectra of antibody-conjugated fluorophores and fluorescent dyes. When comparing the spectra of 4′,6-diamidino-2-phenylindole (DAPI) and Alexa Fluorophores in BABB, Scale, and PBS, we found differences in both wavelength and intensity of the emitted light. When individual antibodies are excited separately, the spectral shift does not impair the optical analysis in most common filter sets, as the spectral shifts are relatively small. Contrary, in high throughput-techniques several wavelengths are often excited simultaneously using multiband pass filters with much tighter tolerances for spectral overlap. In this case, the spectral shifts caused by clearing may exacerbate cross talk. Here, we describe clearing-induced alterations in both wavelength and intensity of fluorescent emission spectra and analyse possible impairments of immunostaining experiments due to unexpected spectra caused by clearing reagents.

## Results

Accurate quantification of fluorescent signals relies on the orchestrated interplay of fluorophores, light sources, optical filters, and detectors. Most biological imaging hardware has been optimized to accurately detect well-characterized fluorophores in aqueous solutions. In our analysis, we use both platform-agnostic plate readers as well as example scanning confocal setups as representative imaging systems.

### BABB alters the emitted fluorescent intensity of fluorophores and fluorescent dyes

**Figure 1:**
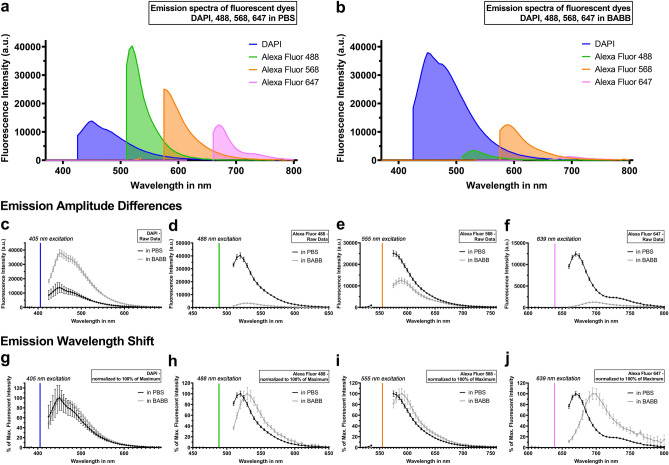
BABB alters the fluorescent emission spectra of fluorophores and fluorescent dyes. Plate reader-based emission spectra of Alexa Fluorophores and DAPI in PBS (**a**) vary from those in BABB (**b**). The line graphs (**c**–**j**) represent the individual emission signals shown in (**a**,**b**): Raw emission data visualize the differences in the amplitude between measurements in PBS (black line) and BABB (grey line, **c**–**f**). Emission data normalized to their peak intensity highlight the shift of the emission peaks to longer wavelengths (**g**–**j**). DAPI was excited at 405 nm (**c**), Alexa Fluor 488 at 488 nm (**d**), Alexa Fluor 568 at 555 nm (**e**), and Alexa Fluor 647 at 639 nm (**f**)**.** In order to visually distinguish individual spectra, the gaps resulting from missing data points (see “Methods” section) were closed by vertical lines. Error bars indicate standard deviation. n = 8 independent wells for each datapoint.

Comparing plate reader-based, cell-free emission spectra of fluorophores in the organic BABB clearing-solution to those in aqueous PBS, BABB decreased the emitted light intensity of Alexa Fluors 488, 568, and 647 compared to PBS (Fig. 1a–f). Fluorescent peak intensity values were two to eightfold weaker compared to the signal in PBS. Contrary, the peak intensity of the fluorescent dye DAPI was increased by approx. 2.5-fold compared to PBS (Fig. 1a–c).

### BABB shifts the fluorescent emission spectra of fluorophores to longer wavelengths

In addition to altered amplitudes in the emission spectra, BABB also shifted emission peaks to longer wavelengths (Fig. 1g–j). In particular, the cell-free emission curves of Alexa Fluors 488, 568, and 647 in BABB demonstrated an excitation-wavelength-dependent bathochromic shift ranging from 10 to 25 nm. Longer excitation wavelengths resulted in a more pronounced right shift of BABB-based emission peaks compared to aqueous conditions. In contrast to Alexa Fluor secondary antibodies, the peak emission wavelength of the fluorescent dye DAPI was not affected by the clearing solution (Fig. 1g).

### BABB alters emission spectra of fluorophores in fixed and stained tissues

**Figure 2:**
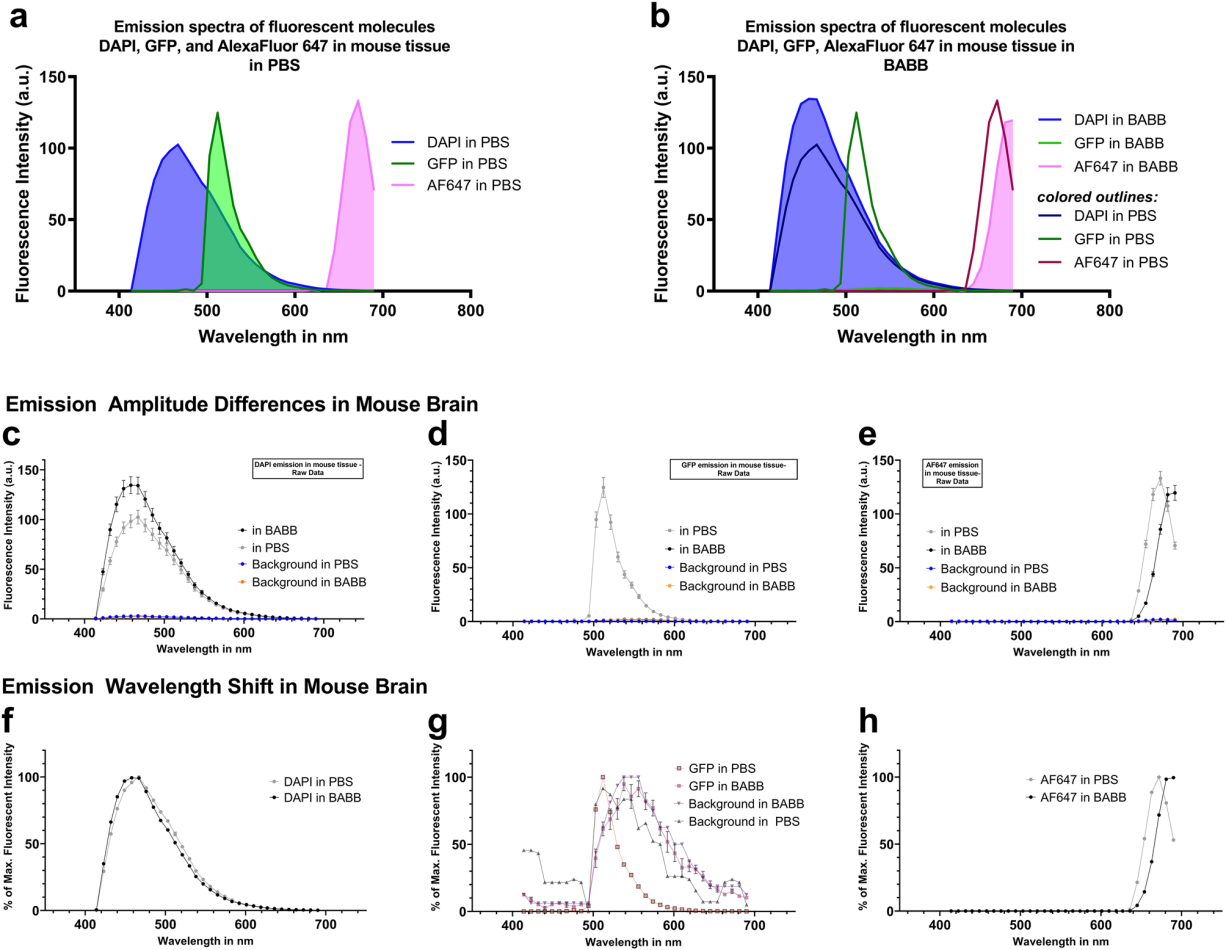
BABB alters the fluorescent emission spectra of fluorophores and fluorescent dyes in mouse brain tissues. Confocal microscopy-based emission spectra of Alexa Fluorophore 647, eGFP, and DAPI in PBS (**a**) vary from those in BABB (**b**). BABB quenches native eGFP fluorescence to background levels (**a,b,d,g**). Colored outlines in (**b**) represent emission profiles of PBS shown in (**a**) to facilitate visual comparison. The line graphs (**c**–**h**) represent the individual emission signals of BABB-cleared tissues shown in (**a**,**b**): Raw emission data visualize the differences in the amplitude between measurements in PBS (grey line) and BABB (black line) (**c**–**e**). Emission data normalized to their peak intensity highlight the shift of the emission peaks to longer wavelengths (**f**–**h**). DAPI was excited at 405 nm, eGFP at 488 nm, and Alexa Fluor 647 at 633 nm**.** Error bars indicate standard error of the mean. Spectra are shown as means measured in n = 16 regions of interest per fluorophore across 4 separate biological samples.

Results obtained in cleared and non-cleared mouse brain tissues expressing eGFP-tagged nuclear histones confirmed spectra obtained in cell-free plate reader experiments (Fig. 2a–h). As previously published^12,27^, BABB-based and other aqueous and solvent-based clearing methods^28,29^ can strongly quench the fluorescence of commonly used proteins, including eGFP. Thus, the fluorescence intensity of eGFP in BABB-cleared samples dropped to background levels (Fig. 2a,b,d,g). To counteract the signal loss and still visualize transgenic fluorescent proteins in these scenarios, most protocols perform immunostaining against the fluorescent protein of interest, coupling a specific antibody to the protein, which either carries a directly conjugated dye or is amplified via a secondary antibody along with a conjugated dye. Using the latter strategy with Alexa Fluor 647-coupled secondary antibodies as the dye with the greatest right shift from our cell-free experiments, we confirmed a spectral right shift in BABB as encountered before (Fig. 2h). We also observed an attenuation of the 647 signal, albeit at a smaller magnitude than in plate reader experiments (Fig. 2e). In contrast, the amplitude of DAPI was increased by BABB clearing (Fig. 2c), though no spectral shift was observable (Fig. 2f), which is consistent with the earlier plate-reader results.

### BABB may exacerbate cross talk of simultaneously excited fluorophores and fluorescent dyes

**Figure 3:**
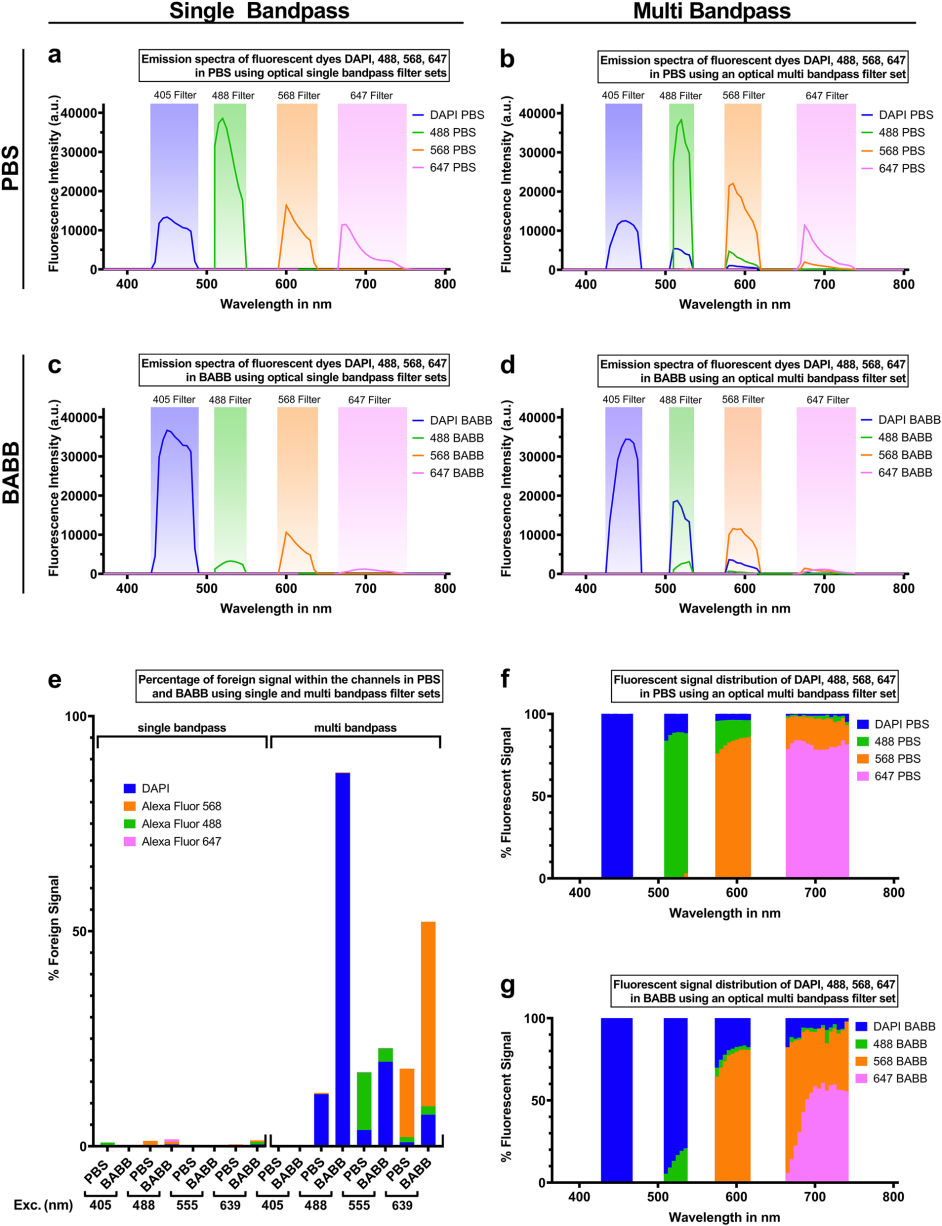
The BABB-induced spectral alterations exacerbate crosstalk in multiband excitation scenarios. Single excitation and single bandpass emission spectra barely contain foreign signal in PBS and BABB (**a**,**c**,**e**). The foreign signal is caused by fluorophores which emit light in another filter range. Multi excitation and multi bandpass emission spectra contain a noticeable amount of foreign signal (**b**,**d**,**e**). Relative contributions of each fluorophore per emission filter band differ strongly between PBS and BABB (**f**,**g**). The foreign signal in multiband settings exceeds that in single bandpass setups (**e**). The line graphs represent the emission spectra of excited fluorophores and fluorescent dyes in PBS (**a**,**b**) and BABB (**c**,**d**) using optical single (**a**,**c**) and multi (**b**,**d**) bandpass filter sets indicated by shaded areas. DAPI (blue), Alexa Fluor 488 (green), Alexa Fluor 568 (orange), and Alexa Fluor 647 (magenta) are excited individually (single bandpass) or simultaneously (multi bandpass) with 405 nm, 488 nm, 555 nm, and 639 nm light. The bar graph (**e**) compares the percentage composition of the foreign signal within certain emission bands (filter bands illustrated in **a**–**d**) using single and multi-bandpass filter sets in BABB and PBS. This reflects the percentage of light that could be misattributed to an incorrect fluorophore in certain scenarios. The stacked graphs illustrate the fluorescent signal distribution within certain emission bands using optical multi bandpass filter sets in PBS (**f**) and BABB (**g**). n = 8 independent wells and measurements for each datapoint. Error bars were omitted for clarity, but are the same as in Fig. 1.

Common biological microscopes include optical filter sets, which limit the emission spectra to particular emission bands to provide spectral selectivity for distinct fluorophores and to reduce interference by autofluorescence. With single wavelength excitation in combination with narrow single band pass filters, most common microscope filter sets resolve emission signals into their correct emission bands despite the shifts caused by clearing (Fig. 3a,c). However, in high throughput scenarios, several spectrally distinct antibodies are often excited simultaneously using multiband pass filters in order to reduce acquisition times, and under these conditions the emission signals may not be resolved into their correct emission bands due to the clearing-induced shifts in amplitude, and, to a lesser degree, shifts in wavelength (Fig. 3b,d). Quantitatively, the BABB-induced shifts of emission wavelength and strength starkly raised the channel crosstalk between fluorophores and fluorescent dyes in multiband pass applications (Fig. 3e–g). In the presence of BABB, the crosstalk of DAPI was increased by 86.8% in the 488 nm filter range and by 19.7% in the 568 nm filter range. Some fluorescence emitted by the Alexa Fluor 488 was incorporated into the emission spectrum of Alexa Fluor 568 contributing 3.1% to the total signal. Moreover, Alexa Fluor 568 added to the fluorescence emission spectrum of Alexa Fluor 647 and increased the crosstalk by 42.8% compared to aqueous conditions.

### Clearing-based shifts in amplitude and wavelength depend on the individual combination of fluorophore, cellular environment, and clearing agent

**Figure 4:**
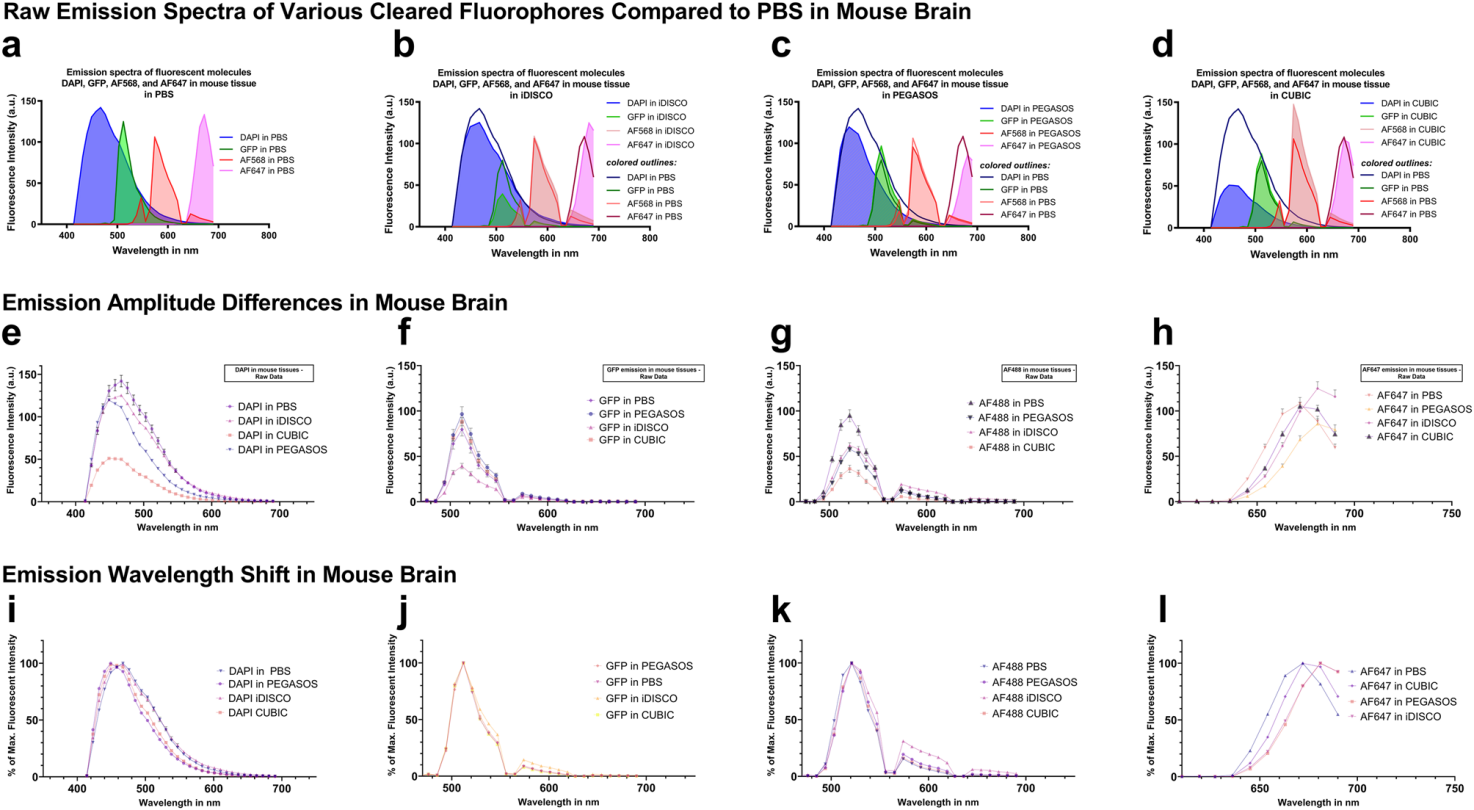
iDISCO, CUBIC, and PEGASOS clearing alter the fluorescent emission spectra of fluorophores and fluorescent dyes in mouse brain tissues. Confocal microscopy-based emission spectra of Alexa Fluorophores 488, 568, 647, eGFP, and DAPI in PBS (**a**) vary from those in iDISCO (**b**)**,** PEGASOS (**c**)**,** and CUBIC (**d**). Colored outlines in b-d represent emission profiles of PBS shown in a) to facilitate visual comparison. The line graphs (**e–h**) represent the individual emission signals of cleared tissues shown in (**a**) through (**d**) with Alexa Fluorophore 488 in addition: Raw emission data visualize the differences in the amplitude between measurements in PBS and cleared tissues (**e**–**h**). Emission data normalized to their peak intensity highlight the shift of the emission peaks to altered wavelengths (**i**–**l**). DAPI was excited at 405 nm, eGFP and Alexa Fluor 488 at 488 nm, and Alexa Fluor 647 at 633 nm**.** Error bars indicate standard error of the mean and are at times too small to be visible. The reduction of fluorescence emission around 560 nm stems from the beam splitter in the light path (**f**–**h** and **j**–**l**; **e**,**i** used a different beam splitter without this gap). Spectra are shown as means measured in n = 25 regions of interest per fluorophore across 5 separate biological samples.

**Figure 5:**
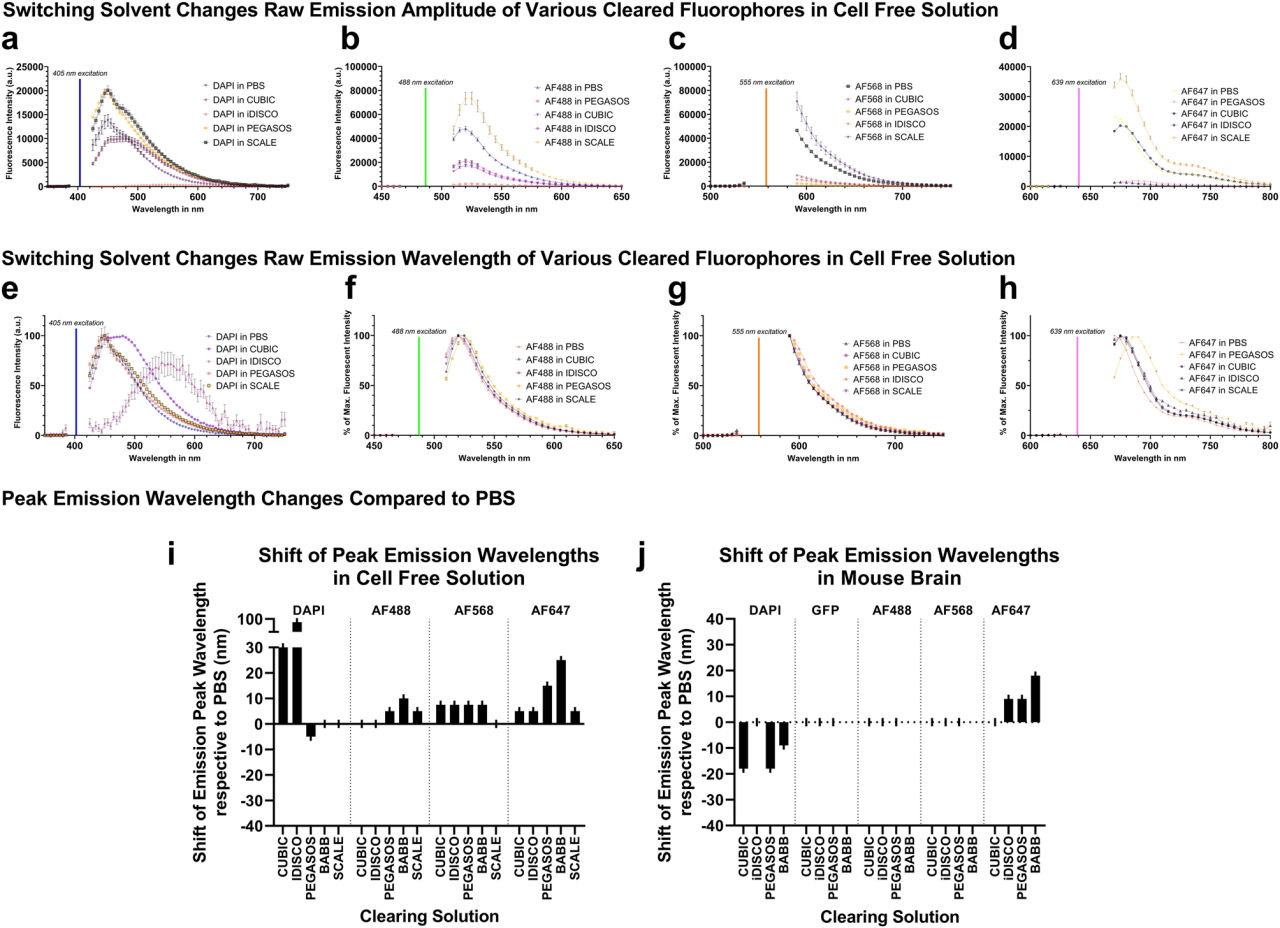
iDISCO, CUBIC, SCALE, and PEGASOS clearing alter the fluorescent emission spectra of fluorophores and fluorescent dyes in cell-free solutions. Raw plate reader-based emission spectra visualize the differences in the amplitude between measurements in PBS and iDISCO, CUBIC, SCALE, and PEGASOS clearing (**a**–**d**). Emission data normalized to their peak intensity highlight the shift of the emission peaks to altered wavelengths (**e**–**h**). Bar graphs summarize the direction and magnitude of emission peak wavelength shifts per fluorophore and clearing agent in cell-free solutions (**i**) and in mouse brain tissue (**j**). Bar graphs visualize the shifts depicted in Figs. 4 and 5 and represent the shifts of average emission curves generated from at least n = 8 independent measurements and wells per datapoint. DAPI was excited at 405 nm (**a,e**), Alexa Fluor 488 at 488 nm (**b,f**), Alexa Fluor 568 at 555 nm (**c,g**), and Alexa Fluor 647 at 639 nm (**d,h**). The gaps in spectra surrounding the excitation wavelength are due to technical limitations in the plate reader (see “Methods” section). Error bars indicate standard deviation. n = 8 independent wells for each datapoint.

Clearing protocols vary widely in their composition of solvents, drying agents, surfactants, and buffers. To broaden our coverage of the findings with BABB-based clearing, we also examined fluorescence emission spectra of PEGASOS (based on benzyl benzoate and polyethylene glycol) and iDISCO (based on dibenzyl ether) in the context of mouse brain tissue (Fig. 4) and in cell-free solutions (Fig. 5). As iDISCO, CUBIC, and PEGASOS did not quench GFP fluorescence, we measured the effects of clearing on GFP spectra (Fig. 4f,j), where peak emission wavelength remained unaltered. In parallel, we quantified the effects of water-based clearing protocols CUBIC and ScaleS4^26^ (Figs. 4, 5) to explore clearing approaches that do not rely on organic solvents. Overall, we identified several factors that influence the amount and direction of spectral shift (for an overview, please see Fig. 5i,j):

- the presence or absence of tissue. For example, CUBIC resulted in a bathochromic shift of 30 nm for the emission peak of DAPI in cell-free solution; however, CUBIC caused a hypsochromic shift of 20 nm in mouse brain. Also, almost all clearing protocols caused a bathochromic shift of 5–10 nm for Alexa Fluor 568 in cell-free conditions, but not in mouse brain.
- the identity of the fluorophore. For example, whereas DAPI and Alexa Fluor 647 undergo spectral shifts upon clearing in mouse tissue, GFP and Alexa Fluor 488 do not. Also, fluorescent beads shifted peak emission frequencies differently than antibody-conjugated fluorophores under the same conditions, i.e. iDISCO shifted bead emission at 488 nm excitation, but did not cause any shift in cell-free or tissue samples under equivalent circumstances (fig. S1, cf. Fig. 5i).
- the clearing reagent. For example, whereas iDISCO, PEGASOS, and BABB caused a bathochromic shift of 10–20 nm for Alexa Fluor 647, CUBIC did not cause any peak shift.

Generally, cell-free conditions promoted chromic shifts of peak emission, with most clearing agents and fluorophores altering peak emission wavelengths in comparison to phosphate-buffered saline (PBS) controls (5 conditions out of 20 did not shift in cell-free solutions compared to 12 out of 20 conditions in mouse tissue).

Emission amplitudes varied widely between different clearing regimens. For example, CUBIC peak emission amplitude for DAPI was 30% that of PBS under equivalent conditions in mouse tissue (Fig. 4e). In cell-free setups, PEGASOS severely limited emission amplitude compared to PBS controls (emission amplitude lower by a factor of > 10 in Alexa Fluor 488 (Fig. 5b) and Alexa Fluor 568 (Fig. 5c). In mouse brain, however, PEGASOS did not attenuate the emission as much (50% signal reduction compared to PBS with AF488 (Fig. 4g)). At times, in tissues, PEGASOS even provided the highest emission levels, surpassing PBS controls (20% higher GFP emission than PBS controls (Fig. 4f)).

The discrepancies of emission peak wavelength and amplitude did not stem from differences in refractive index of the clearing solutions. AF647 in a serial dilution of sucrose in PBS did not result in chromic shifts and caused only very minor emission amplitude differences (> 10% of the overall signal) that do not explain the shifts observed in clearing solutions (fig S2). The chromic shifts induced by each clearing solution also did not correlate well with refractive index values (R^2^ values of a linear regression below 0.49 for cell-free and tissue-based data, slopes were not significantly different from zero) (fig. S3).

A number of publications relate solvent polarity to their ability to induce chromic shifts (for review, see^30^). Were chromic aberrations after clearing solely due to solvent characteristics, one would expect a correlation between solvent polarity (for example the molar electronic transition energies E_T_(30) according to^31^) and the magnitude of the chromic shift. The chromic shifts in neither cell-free nor mouse tissues correlated well with solvent polarity (fig. S3, R^2^ = 0.02 cell-free, R^2^ = 0.21 for mouse tissue). However, the presence of mouse tissue clearly altered the effect of solvents on chromic shifts (also cf. Fig. 5i,j).

Consistent with solvatochromic effects, the addition of clearing solutions to Alexa Fluor 647 resulted in a bathochromic shift of the absorption peak wavelength up to 15 nm (Fig. S5).

Taken together, these findings reinforce the interdependence of the three key constituents in clearing approaches: clearing solutions, fluorophores, and tissue components.

### Clearing-induced spectral shifts can alter the quantitative assessment of multiband excitation experiments

**Figure 6:**
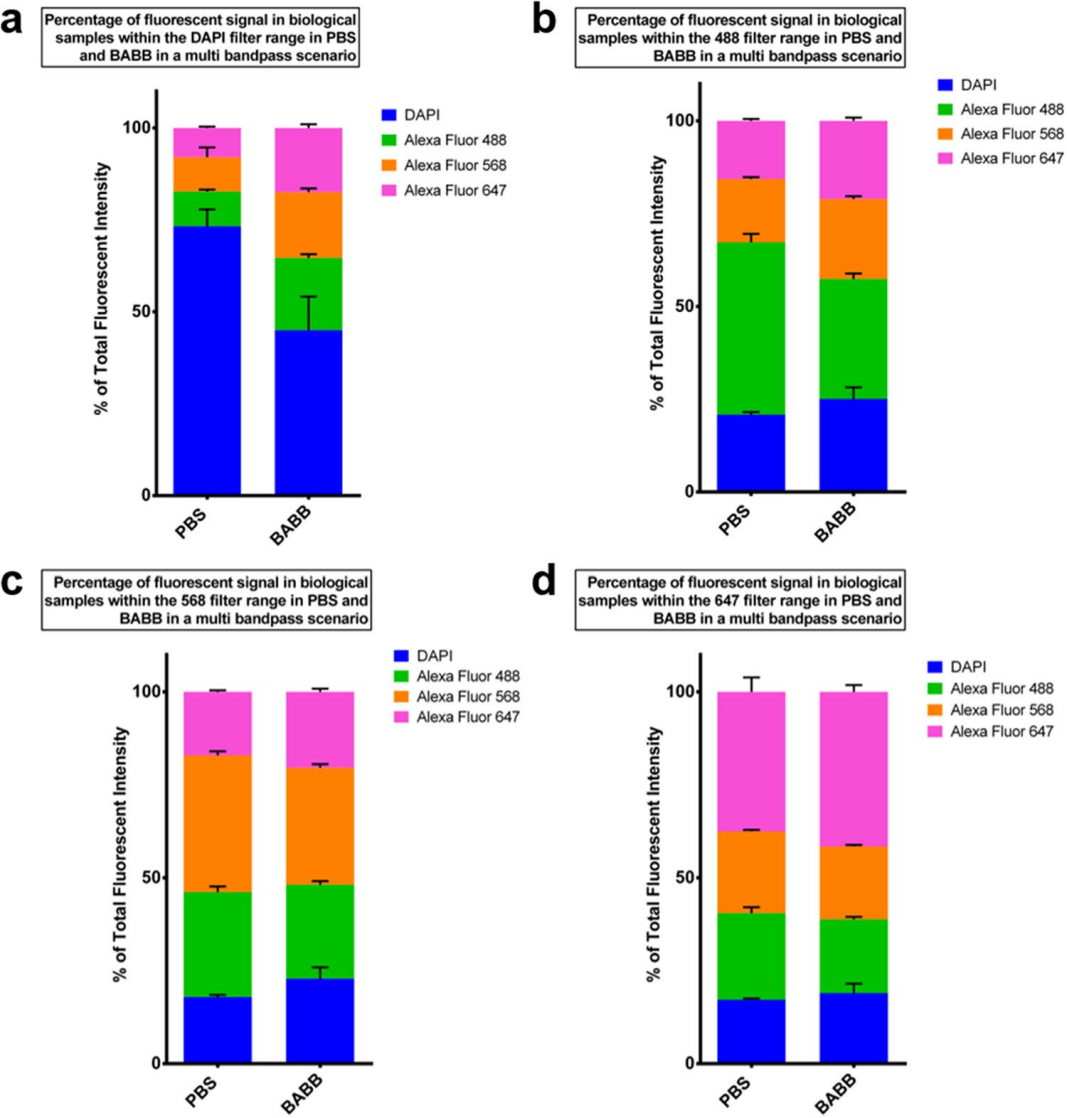
The BABB-induced spectral alterations exacerbate crosstalk in multi-bandpass excitation scenarios in biological samples. BABB clearing exacerbates crosstalk in biological samples in the DAPI (**a**) and the 488 Filter range (**b**) in multi bandpass scenarios, which is shown by the reduced percentages of total DAPI and Alexa Fluor 488 signal intensities for BABB in the respective filter ranges. The stacked graphs illustrate the total fluorescent signal distribution of DAPI (blue), Alexa Fluor 488 (green), Alexa Fluor 568 (orange), and Alexa Fluor 647 (magenta) in PBS (left) and BABB (right) in biological samples (MEFs). Data are grouped by emission filter wavelength: in the DAPI filter range (**a**), the 488 nm filter range (**b**), the 568 nm filter range (**c**), and the 647 nm filter range (**d**). Samples were immunostained with an anti-GAPDH primary antibody, and respective secondary antibodies, or DAPI, and measured with a high content imaging system. Error bars indicate standard deviation. n = 8 independent wells and measurements for each datapoint.

To further investigate spectral alterations in multiband excitation scenarios, we also performed fluorescent immunostaining of mouse embryonic fibroblasts (MEFs) in BABB and PBS (Fig. 5). As in mouse brain tissue, we found similar trends to those seen in cell-free samples in the DAPI and 488 nm filter ranges confirming the effects, albeit with a lowered magnitude. Generally, under BABB conditions, the amount of correctly attributable signal decreases while signal from spectrally adjacent fluorophores increases. For example, in the DAPI filter range, DAPI accounts for 75% of the signal in PBS, but only 45% in BABB, with the percentage of 488 signal approaching 20% (Fig. 6a). A reduction of emission-band-specific (and thus correctly attributable) signals can be observed for the other emission filter ranges as well (Fig. 6b,c), with the exception of the 647 filter range, where the signal of the 647 nm fluorophore represents a slightly larger percentage of the incident light in BABB compared to PBS (Fig. 6d).

## Discussion

Optical clearing enables analysis of hitherto non-accessible/opaque tissues and has enabled better understanding of 3D tissue architecture^32^. With 3D cell models on the rise, clearing will likely play an increasingly important role in fluorescent analysis of whole mount immunostained tissues. However, the chemicals that homogenize refractive indices and reduce optical scattering come at a price: they may alter key physical attributes of fluorophore light emission and absorption and may thus influence experimental results.

Solvent-based spectral shifts of fluorescence are a phenomenon known as solvatochromism^33^, and can be either positive (more polar solvents causing a blue-shift in the emission spectrum), or negative (more polar solvents causing a red shift). In either case, the dielectric properties of the solvent and a differing capacity for hydrogen bonding change the ground and excited states of fluorophores. This results in an altered energy gap between the two states, which in turn alters the energy and wavelength of emitted photons. While these shifts are not always desirable for all imaging applications, in principal, they can be quite advantageous: For GFP, systematically altering the hydrogen-bonding capacity of key protein residues to the central chromophore is one of the strategies that has allowed the development of fluorescent proteins spanning the visible wavelength spectrum^34–38^. Indeed, most fluorophores exhibit some degree of solvent-based spectral alteration^39–44^, making this issue relevant for a large variety of experimental conditions and setups. Here, the addition of clearing solutions caused a bathochromic shift in both emission and absorption spectra (fig. S5), consistent with the involvement of solvatochromism in spectral changes during clearing.

A number of questions remain. Why do chromic shifts differ substantially between cell-free and tissue-based samples? Solvatochromism is not limited to solvent-fluorophore interactions. Reichardt et al.^31^ quotes an unnamed reviewer suggesting solvatochromism should rather be called “peri-chromism” from the Greek “peri” meaning “around”, as energy states of fluorophores can be altered by many interactors, be they liquid, solid, or gas, as long as they affect the rate change of energy transition states of a given molecule of interest. Thus, chromic shifts can be influenced by more than solvents alone, suggesting that the complex mixture of biochemicals in cells can contribute to spectral shifts. This sensitivity of fluorophores has in fact been utilized to detect subtle changes in their immediate chemical surrounding^45–47^.

In our work, generally the magnitude of chromic shifts was reduced in tissue compared to cell-free solutions (Fig. 5i,j). This could be due to the stabilizing effects of local tissue moieties on the fluorophore. In cell-free solutions, many fewer agents can buffer local changes in charge or pH or mitigate aberrant hydrogen bonding. Given the general sensitivity of fluorophores to their immediate molecular environment, it is not surprising that our data diverge between cell-free and tissue-based findings. Clearly, the local environment in tissues contains a wealth of additional components, each of which can interact with fluorophores before, during, and after excitation and relaxation, thus changing the energy band gap and resulting photon wavelength.

Our results show the co-dependence of the emission shift on molecular details of solvents, fluorophores, and the presence of cells. Especially the effects of clearing on emission amplitude are surprising. Whereas cell-free setups tended to significantly reduce fluorescence upon clearing (for examples see Fig. 5b), the same clearing solutions quenched fluorophores less, or even boosted light emission in tissues (for example PEGASOS in Fig. 4f). This apparent boost may stem from biological components present in cells that mitigate solvent-induced quenching, but details remain speculative.

Our data suggest that the alterations to emission wavelength likely have complex origins. Plotting chromic shifts versus refractive indices of different clearing solutions (fig. S3) or the molar electronic transition energy E_T_(30) as a measure of solvent polarity^31^ (fig S4) did not provide evidence of a clear correlation. The E_T_(30) used here is an empirically derived measure of polarity that attempts to take into account many of the more complex molecular interactions that may not be fully understood at the theoretical level (details in^31^). For clearing solutions, the situation gains additional complexity because they contain a multitude of ingredients and calculating effective solvent polarity for mixtures, even much simpler ones than used for clearing (not to mention the myriad biochemicals contained in tissues), is anything but straightforward^30,48^. Here, we approximated solvent polarity of clearing solutions by simply considering the major constituent in the final clearing solution. The authors understand that this does not reflect the physical reality of the samples, but our emphasis in this work is not centered on the theoretical underpinnings of solvent-fluorophore interactions, but rather a practical one: Researchers need to be aware that the emission spectra they expect may not be the emission spectra they get when they employ clearing techniques.

On the practical side, our data demonstrate that BABB and Scale-based clearing techniques will not significantly alter quantitative results based on *single* excitation filter sets but will exacerbate crosstalk and potential signal misattribution in *multiband* excitation scenarios. This may be of particular importance for high content campaigns. Since a number of popular clearing solutions—organic solvent-based BABB, PEGASOS, iDISCO, and water-based CUBIC and Scale—induce spectral alterations, other clearing techniques may also be affected. Consequently, researchers relying on clearing in quantitative multiband excitation experiments should crosscheck their fluorescent signal after clearing in order to inform the proper selection of filter sets and fluorophores for analysis.

In this study, all fluorophores were analysed individually and in separate wells or samples, not considering any potential co-excitation/interaction between fluorophores. In daily laboratory practice, tissue samples often contain multiple fluorophores with overlapping emission and excitation spectra, thus the potential for channel crosstalk could be even higher than outlined here.

Modern microscopes allow emission fingerprinting to untangle the crosstalk of different signals in multicolour imaging. With this method, overlapping emission profiles are separated digitally using algorithms based on characteristic reference spectra. However, the standard reference spectra obtained in PBS may not represent the spectra of fluorophores in clearing solutions. Consequently, correct spectral attribution requires specific reference data for every single clearing protocol and fluorophore. Moreover, clearing-induced spectral alterations in emission magnitude and frequency can increase the proportion of foreign signals within a channel to such an extent that a different but adjacent emission profile is completely masked (Figs. 1b, 2d). This scenario could lead to a misattribution of the fluorescent signal to the stronger emitter, with the weaker signal irretrievably lost in the broad interference band of the stronger fluorophore.

For optimal quantitative fluorescent assessment of cleared tissues, we recommend several strategies. These share a number of points with common good practices for fluorescent microscopy but could be even more crucial with clearing.

1. Obtain full spectrum absorption and emission spectra of your fluorophores in clearing solutions to make informed decisions about filter sets and fluorophore selection before conducting crucial experiments.
2. Select fluorophores that have distinct emission spectra spaced far apart in the emission spectrum under clearing conditions.
3. Use single band excitation with narrow band emission filter sets. This will lengthen acquisition times, but may result in more accurate quantification.
4. Use online fingerprinting to resolve spectra, but make sure to use custom-tailored reference spectra for samples in clearing solution.
5. For high throughput campaigns, measure and optimize spectra in cleared conditions, and consider obtaining custom multiband emission filters with narrower bands corresponding to the new peak wavelengths in clearing conditions, thus minimizing crosstalk in cleared samples. In the context of larger screening campaigns, filters do not add significant cost and can help resolve spectra to the correct fluorophore.

We hope that these results help other researchers to generate fluorescent data with confidence, resulting in highly quantitative, high quality results that drive biological insight in 3D cell culture.

## Methods

### Fluorescence measurement of fluorophores and fluorescent dyes in solution

We diluted Alexa Fluor donkey anti-rabbit secondary antibodies (Thermo Fisher) and nuclear counterstains Hoechst 33342 (Thermo Fisher Scientific) and DAPI (Sigma-Aldrich) in phosphate buffered saline (PBS) (Sigma-Aldrich), 1:1 (v/v) benzyl-alcohol/benzyl benzoate (BABB) (Sigma-Aldrich), or ScaleS4^26^, CUBIC, PEGASOS, and iDISCO (compositions see below): Alexa Fluor 488 (1:1000, Thermo Fisher Cat. No. #21206), Alexa Fluor 568 (1:200, Thermo Fisher Cat. No. #A10042), Alexa Fluor 647 (1:200, Thermo Fisher Cat. No. #A31573), final concentration 0.5 μg/mL DAPI, and final concentration 0.5 μg/mL Hoechst. Here, we adjusted the concentration of each secondary antibody to keep the fluorescent signals within the detectable range of the plate reader and ensure similar signal strength of all antibodies. We supplemented samples containing only DAPI or Hoechst with 12.5 ng/µl plasmid DNA (as they only fluoresce in contact with DNA), mixed them thoroughly, and transferred 200 µl of the solution to an organic solvent-resistant 96-well flat-bottom cyclic olefin copolymer (COC) plate (Greiner Bio-One). We detected the fluorescent emission and absorption spectra with a SynergyMx plate reader (BioTek). ScaleS4 consisted of D-(–)-sorbitol 40%, Glycerol 10% (Roth), Urea (4 M), Triton X-100 0.2%, Dimethylsulfoxide 20%, all data given in w/v in PBS, reagents from Sigma-Aldrich unless noted otherwise. Final solution for iDISCO was dibenzyl ether (Sigma), for PEGASOS it was a mixture of benzyl benzoate-polyethylene glycol (BB-PEG) clearing solution (75% benzyl benzoate (v/v) (fisher scientific) + 25% PEG-MMA-500 (v/v) (Sigma). CUBIC clearing cocktail consisted of reagent-2 (50 wt% sucrose (Sigma), 25 wt% urea (Sigma), 10 wt% triethanolamine (Sigma)).

Absorption spectra were generated in the same clearing solutions and plate reader, but antibody dilution was raised to 1:100 for a better s/n ratio.

Fluorescent beads (sicastar-greenF and sicastar-redF, micromod) were diluted 1:1000 from stock to 1 ug/ml in a 1:2 serial dilution of sucrose (Merck) in PBS, starting at 30% w/v. PBS and 30% sucrose without added beads served as controls.

### Processing of fluorescence measurement data

As a straightforward experimental setup designed to minimize channel crosstalk, one sample corresponding to one well contained only one fluorophore or dye resulting in a single emission spectrum. We displayed these single spectra in the same diagram in Figs. 1a,b, 2a,b, 3a–d, and 4a–d to allow better visual comparison side-by-side.

We performed all fluorescence measurements with > n = 3 replicates (detailed n’s given in the figure legends) and processed the raw data from the plate reader with Microsoft Excel (Microsoft Office Professional Plus 2016). First, the mean background (PBS, BABB, CUBIC, iDisco, PEGASOS, or Scale without secondary antibodies or fluorescent dyes) was subtracted from the fluorescent intensity value of each sample. If this resulted in a negative value, the intensity was set to 0 (arbitrary units). We illustrated the mean fluorescent intensities at each wavelength in area diagrams (Figs. 1 and 5). Since the plate reader cannot acquire emission data within 20 nm of the excitation wavelength, the missing data points result in gaps within the diagrams. These were closed by vertical lines.

We also graphed the same data in individual line diagrams to demonstrate amplitude differences between emission spectra in PBS and BABB, CUBIC, iDisco, PEGASOS, or Scale (Figs. 1c–f and 5a–d). The error bars indicate the standard deviation or error of the mean to facilitate visibility of the error bars; exact nature of error bars given in each figure legend.

We further processed the mean fluorescent intensity values to mimic the effect of optical single and multi bandpass filter sets of a confocal laser scanning microscope. To this end, we multiplied the mean fluorescent intensities with the percentage transmission of the single bandpass filters (405 Filter: 49000; 488 Filter: 49904; 568 Filter: 49031; 647 Filter: 49914) and multi bandpass filters (Filter: 89401) from Chroma Technology Corp (Fig. 3a–d). The specific percentage transmission values are published on https://www.chroma.com^49–52^.

In order to highlight the channel crosstalk within certain emission bands, we calculated the percentage of foreign signal contributing to the total fluorescent intensity per channel. The foreign signal is defined as the apparent signal of a fluorophore that is recorded as originating from a different fluorophore. Thus, the foreign signal of one or more fluorophores contributes to the total fluorescent intensity within the emission band of another fluorophore. Here, the total fluorescent intensity within a channel was set to 100% and the fluorescent signal distribution is represented in stacked diagrams (Fig. 3f,g).

Moreover, we identified the initial fluorophores contributing to the foreign signal. For this purpose, the total foreign signal within a channel was set to 100%. The percentage of each single fluorophore or dye contributing to the total foreign signal is illustrated in a bar diagram (Fig. 3e).

To show that increased channel crosstalk due to the BABB solution was also present in biological samples, we calculated the total fluorescent signal distribution within each emission band in PBS or BABB (Fig. 6). Therefore, the total fluorescent signal of all fluorophores and fluorescent dyes within each emission band was set to 100%.

Absorption spectra were background-subtracted as outlined above with n = 3 wells per condition. In addition, the data was normalized per solvent so that the signal between 550 and 800 nm spanned from zero to 100 arbitrary units. Error bars represent the standard error of the means.

For fluorescent beads, data was gathered for n = 3 wells per condition and background corrected with the fluorescent mean of three corresponding wells and solvent without fluorescent beads, as outlined above. Error bars are standard error of the mean.

### Generation and maintenance of C3H mouse feeder cells

We obtained mouse embryonic fibroblasts (MEFs) from 13.5 dpc mice (C3H, Charles River). We cultured MEFs at 37 °C and 5% CO_2_ on gelatin-coated 96-well plates (Sarstedt). MEF medium consisted of DMEM low glucose (Sigma-Aldrich) supplemented with 10% FBS Superior (Biochrom), 1% non-essential amino acids (Sigma-Aldrich), and 1% Penicillin–Streptomycin (Sigma-Aldrich) and was replaced every other day.

MEFs were provided by the cell culture facility in house, where they already existed prior to this study. Compliant with the ARRIVE guidelines, the prior MEF production had been registered: ethical approval „Ableitung embryonaler Fibroblasten der Maus (MEFs) für die in vitro Kultur pluripotenter Stammzellen (PSC)“ (permit number 81–02.05.40.2018.067). This approval was issued by the Landesamt für Natur, Umwelt und Verbraucherschutz (LANUV) of the state of North Rhine Westphalia, Germany, in accordance with the German Law (Tierschutzgesetz) and the European Directive 2010/63. Therefore, we confirm that this study is reported in accordance with ARRIVE guidelines.

### Immunofluorescent staining of C3H mouse feeder cells

We seeded 100.000 MEFs per well in an organic solvent-resistant 96-well flat-bottom COC plate. The next day, we fixed the cells with 4% (v/v) paraformaldehyde (Electron Microscopy Sciences) in PBS for 15 min at room temperature followed by three washing steps with PBS, 5 min each. In order to permeabilized the cells and to block unspecific binding sites, we incubated cells with blocking solution (5% FBS (Biochrom), 0.1% Triton X-100 (Carl Roth) in PBS) for 1 h at room temperature. The primary anti-GAPDH antibody (rabbit monoclonal, Cell Signaling, Cat No. #2118S) was diluted in blocking solution, added to the cells and incubated over night at 4 °C. Afterwards, we washed the cells three times with PBS for five minutes each. Donkey anti-rabbit secondary antibodies (Thermo Fisher, details see above) and nuclear counterstains (Sigma-Aldrich) were diluted 1:1000 in blocking solution: Alexa Fluor 488, Alexa Fluor 568, Alexa Fluor 647, final concentration 0.5 μg/mL DAPI, and final concentration 0.5 μg/mL Hoechst. We added the antibody solution to the cells and incubated them for 2–3 h hat room temperature followed by three washes with PBS for five minutes each.

### Fluorescence measurement of immunostained cell samples

We kept fixed and immunostained C3H mouse feeder cells in 200 µl PBS (Sigma Aldrich) or 200 µl benzyl-alcohol/benzyl benzoate (BABB) (Sigma-Aldrich) per well of an organic solvent-resistant 96-well flat-bottom COC plate and detected the fluorescence emission within set emission bands with the high content microscopy system Operetta (PerkinElmer). Further analysis was performed via the Harmony High Content Imaging and Analysis Software Version 4.1 (PerkinElmer)^53^.

### Generation of mouse brain tissue sections

Brain tissues were isolated from one wild-type B6C3F1 and one Histone-H2B:EGFP reporter mouse^54^. The animals were maintained under a 14-h light/10-h dark cycle with free access to food and water. Female mice were housed in groups of up to 4 per cage and male stud mice were housed individually. Mouse husbandry and tissue derivation were performed according to the German Animal Welfare guidelines and approved by the Landesamt für Natur, Umwelt und Verbraucherschutz Nordrhein-Westfalen (State Agency for Nature, Environment and Consumer Protection of North Rhine-Westphalia). Therefore, we confirm that this study is reported in accordance with ARRIVE guidelines.

For sectioning, freshly dissected brain tissues were collected in PBS with calcium, fixed overnight in 4% (v/v) paraformaldehyde in PBS, and immersed in 15% and 30% (v/v) sucrose in PBS at 4 °C for 24 h each. The tissue was then immersed in OCT embedding medium (Tissue-Tek), frozen and sectioned at 20 μm, mounted onto SuperFrost slides (Thermo Scientific), air-dried, and stored at − 80 °C before further use.

### Immunostaining of mouse brain tissue sections

Slides were protected from light throughout the following procedure. Initially, slides were warmed up to room temperature and washed with PBS to remove OCT medium. To avoid spectral contamination for later analysis, no wax pen was used for encircling the sections. The sections were permeabilized with 0.2% (v/v) Triton X-100 in PBS for 5–10 min at room temperature before blocking them with 2% (w/v) bovine serum albumin (BSA) in PBS for 30 min at room temperature. The primary antibody was chicken anti-GFP (ab13970, Abcam) and was applied 1:300 in 1% (w/v) BSA in PBS overnight at 4 °C. After washing with PBS for 3 × 5 min at room temperature, the sections were treated with secondary antibody (Alexa Fluor 647 fluorescent anti-chicken (A-21449, Thermo Scientific)) diluted 1:200 in 1% (w/v) BSA in PBS for 45 min at room temperature. The slides were washed with PBS for 3 × 5 min at room temperature then kept in PBS prior to imaging and clearing. Before clearing, a subset of slides was counterstained for nuclear DNA using 0.5 μg/mL (w/v) DAPI in PBS for 15 min at room temperature.

### Clearing of mouse brain tissue sections

Following the staining procedure, brain sections were processed with either the BABB, iDISCO, PEGASOS, or CUBIC clearing protocols as described previously^22,55–59^.

For BABB, samples were dehydrated via a methanol (Roth) series (25%, 50%, 75%, 90%, 100%, for 15 min each) and then incubated for 30 min in 1:1 (v/v) methanol/BABB. Finally, they were transferred to and kept in 1:1 (v/v) benzyl-alcohol/benzyl benzoate (BABB).

For iDISCO-based tissue clearing, slides were dehydrated in rising concentrations of 50%, 80% and 100% (v/v in dH_2_O) tetrahydrofuran for at least 1 h. Afterwards, slides were treated with dichloromethane for 30 min. For final clearing, the slides were incubated in dibenzyl ether (Sigma) until clear (around 2 h) and then stored in dibenzyl ether at room temperature.

For PEGASOS-based tissue clearing, slides were placed in a series of rising tert-butanol dilutions (30%, 50% and 70% tert-butanol (Sigma) (v/v in dH_2_O), supplemented with 3% Quadrol (Sigma) (w/v)) for at least 30 min each and then in tert-butanol-polyethylene glycol (tB-PEG) solution (70% tert-butanol (v/v) + 30% PEG-MMA-500 (Sigma) (v/v) + 3% Quadrol (w/v)) twice for 1 h. For final clearing, samples were placed into benzyl benzoate-polyethylene glycol (BB-PEG) clearing solution (75% benzyl benzoate (fisher scientific) (v/v) + 25% PEG-MMA-500 (v/v) + 3% Quadrol (w/v)) until transparency was reached and stored in BB-PEG at room temperature.

CUBIC-based clearing^60,61^ was performed by incubating slides in reagent-1A (10 wt% urea (Sigma, U5128), 5 wt% Quadrol (Sigma, 122,262), 10 wt% Triton X-100 (Sigma, X100) in dH_2_O) up to 2 days, while reagent-1A was replaced every 12 h. Afterwards, samples were washed several times in PBS and were then incubated in reagent-2 (50 wt% sucrose (Sigma, S0389), 25 wt% urea (Sigma, U5128), 10 wt% triethanolamine (Sigma, 90,279) in dH_2_O) to achieve final transparency and stored in reagent-2 at room temperature.

### Spectral analysis in immunostained mouse tissue sections

To analyze the spectral characteristics of the different fluorochromes in tissue-based PBS and BABB environments, we used a Zeiss confocal microscope LSM880_34Ch equipped with a 32-channel spectral detector. For the measurement, the lambda mode of ZENblack (Carl Zeiss Microscopy) was used. The hardware was set to 405 nm laser line with the main beam splitter MBS_405 for DAPI/Hoechst, 488 nm laser line with MBS_488 for GFP and 633 nm laser line with MBS_488/561/633 with a detected spectral range of 409 nm to 695 nm in 8.9 nm steps. Laser power was adjusted separately for each laser line and sample type to maximize spectral signal without saturating the detector. Per fluorophore, we analyzed a minimum of 4 separate tissue sections with a minimum of four regions of interest (ROI) per section. ROIs were drawn to sample spectra from either GFP + , Alexa Fluor 647 + , or DAPI + nuclei, with one additional larger ROI collected from cytoplasmic background. This resulted in a minimum of n = 16 ROIs per spectral measurement. Data were exported from ZENblack as tab-separated text files imported to Microsoft Excel 2016 for reformatting, and graphed using Graphpad Prism 9.1.2 (Graphpad Software, Inc.)^62^. GFP-wildtype tissue sections served as negative controls for GFP spectra and immunostaining fidelity. Data are reported as means with standard error of the mean.

## Supplementary Information

Supplementary Information 1.

Supplementary Information 2.

Supplementary Information 3.

Supplementary Information 4.

Supplementary Information 5.

Supplementary Information 6.

## Data Availability

Data generated or analyzed during this study are included in the figures themselves and are available in tabular form upon reasonable request.
