# Supplementary material for: Tissue clearing may alter emission and absorption properties of common fluorophores
Source: Sci Rep. 2022 Apr 1;12:5551. doi: 10.1038/s41598-022-09303-9 (PMC8975997; doi:10.1038/s41598-022-09303-9)
Supplement: Supplementary file 1 — Supplementary Information 1. [file 41598_2022_9303_MOESM1_ESM.docx]

**Supplementary Materials**

**Supplementary Figure Legends**

**Figure S1: Green and orange fluorescent beads do not display spectral shift in CUBIC, PEGASOS, or BABB clearing compared to PBS conditions.**

Raw plate reader-based emission spectra visualize the differences in the amplitude between measurements in PBS and iDISCO, CUBIC, dH2O, BABB, and PEGASOS clearing **(left side)** in green **(top row)** and orange fluorescent beads **(bottom row)**. Emission data normalized to their peak intensity highlight the shift of iDisco emission peaks to altered wavelengths (**right side**).

Green beads were excited at 488 nm **(top row)**, and orange beads at 555 nm **(bottom row)**. The gaps in spectra surrounding the excitation wavelength are due to technical limitations in the plate reader (see methods section). Error bars indicate standard error of the mean but may be too small to be visible. n=3 independent wells for each datapoint.

**Figure S2: Changes in refractive index do not alter emission wavelengths.**

Raw plate reader-based emission spectra visualize the differences in the amplitude between measurements in serial dilutions of 30% sucrose solution in PBS **(left side)** in Alexa Fluor 647 fluorophores. Emission data normalized to their peak intensity highlight the absence of spectral shifts under the same conditions (**right side**).

Alexa Fluor 647 was excited at 639 nm. The gaps in spectra surrounding the excitation wavelength are due to technical limitations in the plate reader (see methods section). Error bars indicate standard error of the mean but may be too small to be visible. n=3 independent wells for each datapoint.

**Figure S3: Chromic shift fails to correlate with refractive indices of clearing solutions.**

Plotting the refractive index of clearing solutions versus the peak emission wavelength of Alexa Fluor 647 under 636 nm excitation does not result in an obvious correlation. Red symbols represent data gathered in mouse tissue (detailed in fig 4) and green symbols represent data from cell-free experiments (detailed in fig 5). Neither group results in a linear regression fit that exceeds a fit of R^2^ > 0.49.

Datapoints represent the shift of the average peak emission profile generated from n=8 individual samples and wells (cell-free) and 25 regions of interest from 5 independent samples (mouse tissue).

**Figure S4: Chromic shift of Alexa Fluor 647 and solvent polarity do not correlate.**

Plotting the molar electronic transition energy of the major component of each clearing solution versus the peak emission wavelength of Alexa Fluor 647 under 636 nm excitation does not result in an obvious correlation. Red symbols represent data gathered in cell-free experiments (detailed in fig 5) and green symbols represent data from cell-free experiments (detailed in fig 4). Neither group results in a linear regression fit that exceeds a fit of R^2^ > 0.21.

Datapoints represent the shift of the average peak emission profile generated from n=8 individual samples and wells (cell-free) and 25 regions of interest from 5 independent samples (mouse tissue).

**Figure S5: Solvent exposure causes bathochromic shifts in absorbance spectra of AF647.**

Light absorption kinetics of AF647 shifted towards longer wavelengths upon contact with BABB, CUBIC, and PEGASOS, but not iDisco. Each line graph was normalized to span from zero to 100 arbitrary units in the wavelengths from 550 to 800 nm. Data represents the mean and standard error of n=3 independent wells and measurements.
