# Supplementary figures and images for: Tissue clearing may alter emission and absorption properties of common fluorophores
Source: Sci Rep. 2022 Apr 1;12:5551. doi: 10.1038/s41598-022-09303-9 (PMC8975997; doi:10.1038/s41598-022-09303-9)

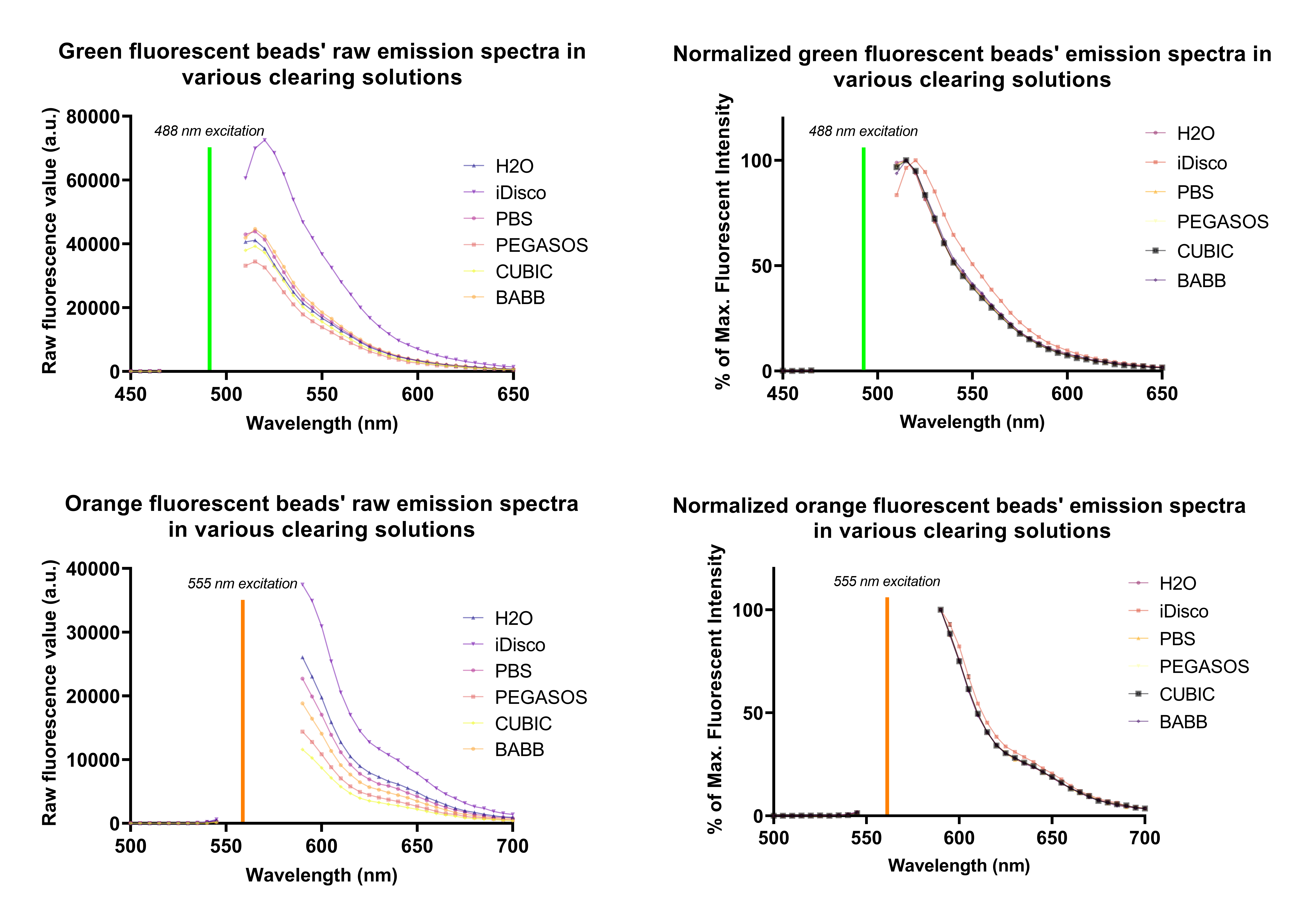

Supplement: Supplementary file 2 — Supplementary Information 2. [file 41598_2022_9303_MOESM2_ESM.png]

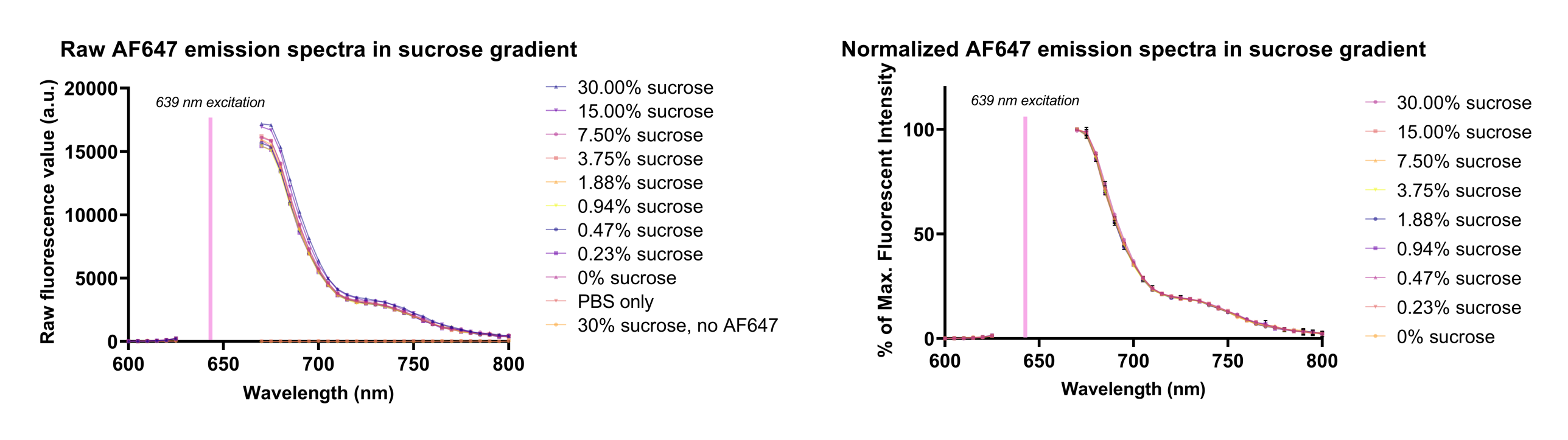

Supplement: Supplementary file 3 — Supplementary Information 3. [file 41598_2022_9303_MOESM3_ESM.png]

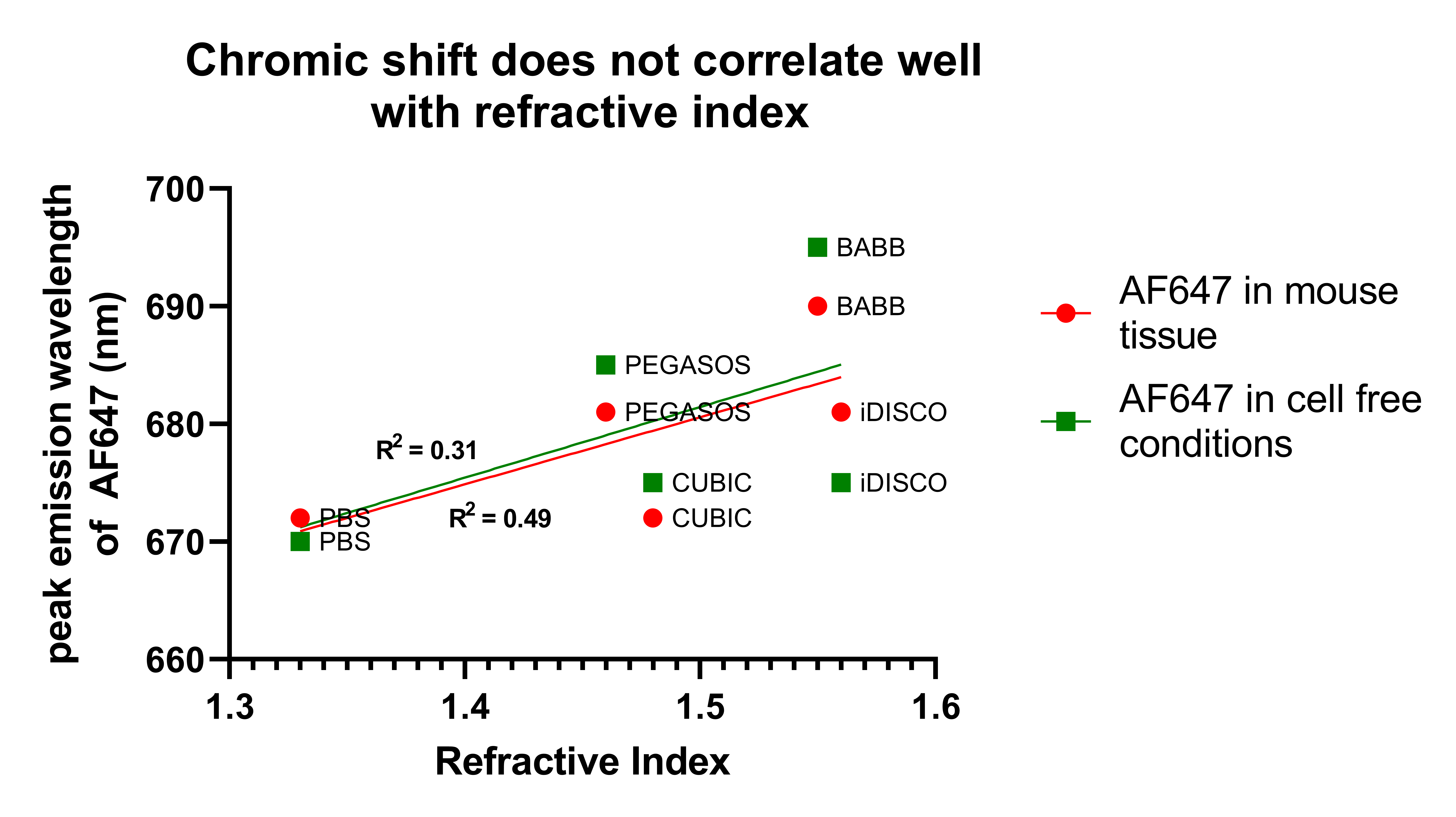

Supplement: Supplementary file 4 — Supplementary Information 4. [file 41598_2022_9303_MOESM4_ESM.png]

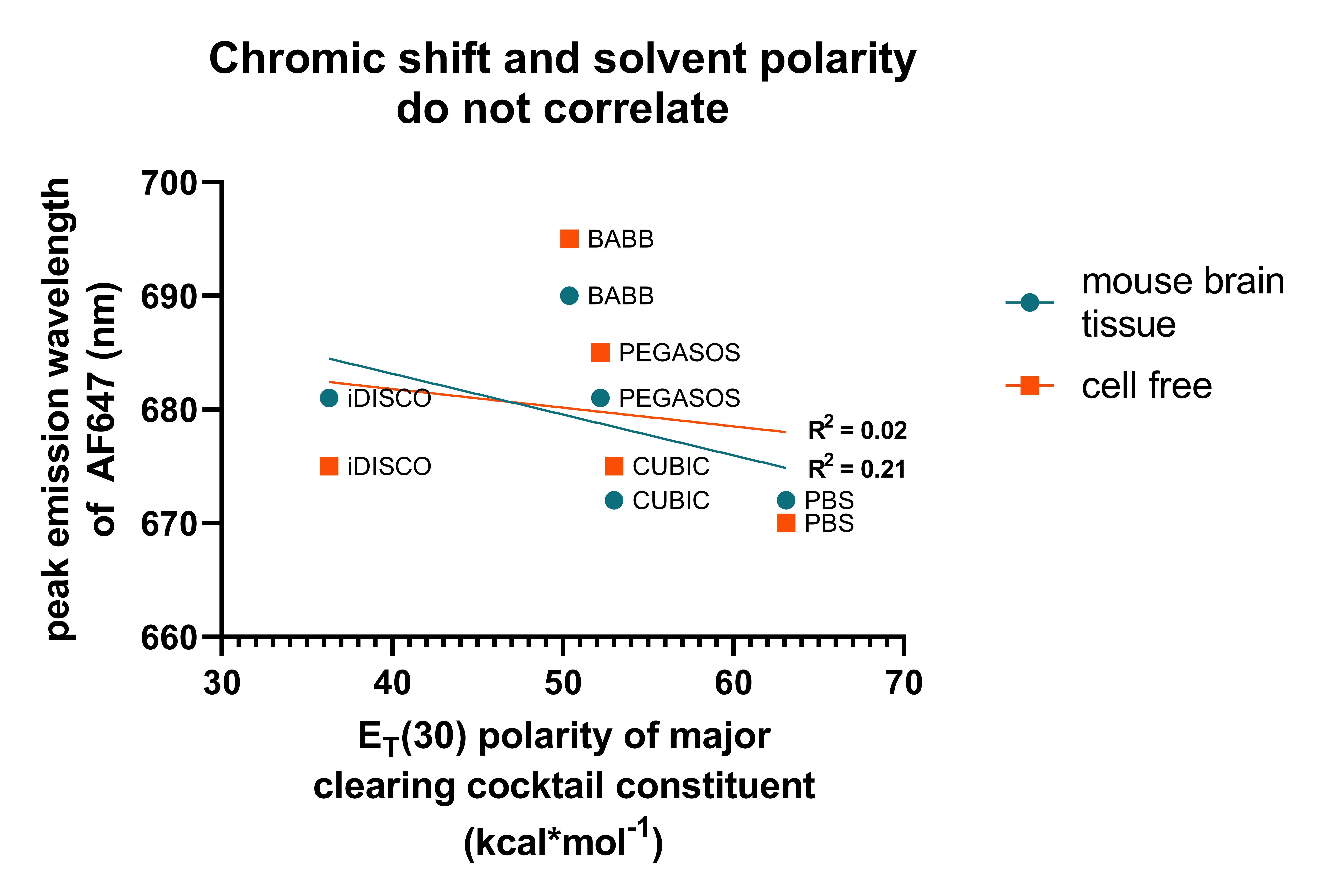

Supplement: Supplementary file 5 — Supplementary Information 5. [file 41598_2022_9303_MOESM5_ESM.png]
